# Paradoxical Massive Colonic Bleeding during Early Response to Upadacitinib in Acute Severe Ulcerative Colitis: A 3-Case Series

**DOI:** 10.1159/iid/adeag004

**Published:** 2026-09-30

**Authors:** Keita Suzuki, Kunio Asonuma, Akira Nogami, Kanade Serizawa, Kenta Nakamura, Taku Kobayashi

**Affiliations:** Center for Advanced IBD Research and Treatment, Kitasato University Kitasato Institute Hospital, Tokyo, Japan

**Keywords:** Acute severe ulcerative colitis, Colonic bleeding, Janus kinase inhibitor, Upadacitinib

## Abstract

**Introduction:**

Acute severe ulcerative colitis (ASUC) is associated with a substantial risk of colectomy. Upadacitinib (UPA) has demonstrated efficacy in ulcerative colitis (UC), and recent studies have suggested its potential use in ASUC. We describe 3 patients with ASUC who developed massive colonic bleeding with hypotension despite early clinical, biochemical, and endoscopic response to UPA.

**Case Presentation:**

All 3 patients had a history of treatment with corticosteroids and infliximab and presented with frequent bloody stools and elevated inflammatory markers at admission. After initiation of UPA 45 mg daily, stool frequency, hematochezia, and C-reactive protein levels rapidly improved. However, after initial resolution of hematochezia, they developed massive colonic bleeding with hypotension, requiring red blood cell transfusion. Colonoscopy at the time of bleeding showed improvement in ulceration and epithelialization of the ulcer base, whereas active bleeding was observed from fragile regenerating mucosa or exposed vessels, requiring endoscopic hemostatic treatment. In 2 patients, bleeding recurred from different sites. Following UPA discontinuation and endoscopic management, no recurrent bleeding was observed any more.

**Conclusion:**

Although gastrointestinal perforation was reported during UPA treatment, colonic bleeding has not been established as a major safety signal in patients with UC. Nevertheless, these cases suggest that massive colonic bleeding during apparent early response to UPA in ASUC may represent a possible treatment-associated event that cannot be fully explained by worsening UC activity alone.

## Introduction

Although most patients with ulcerative colitis (UC) have a mild-to-moderate disease course, approximately 15–25% experience at least one severe flare during the course of their disease, and 10–20% present with acute severe ulcerative colitis (ASUC) at diagnosis [1, 2]. Intravenous corticosteroids are the first-line treatment for ASUC. Current guidelines recommend rescue therapy with infliximab (IFX) or cyclosporine, or surgical intervention, in steroid-refractory cases [3, 4], and ASUC still carries a substantial risk of colectomy [5, 6].

Upadacitinib (UPA) is an oral selective Janus kinase 1 (JAK1) inhibitor. Its efficacy in patients with moderately to severely active UC has been demonstrated in phase 3 trials [7]. Given its rapid onset of action, UPA has recently been used as a therapeutic option for ASUC, including cases requiring rescue therapy [8–10].

Although pharmacovigilance data have identified hematochezia and large intestinal hemorrhage as potential adverse event signals during UPA treatment, these events may have generally been interpreted as worsening of UC activity [11]. The clinical context of these bleeding events remains insufficiently described. Here, we report 3 cases of ASUC in which massive colonic bleeding occurred during apparent early response to UPA, highlighting that such bleeding should not be automatically attributed to worsening UC activity alone.

## Case Presentation

### Case 1

A 50-year-old woman had been diagnosed with extensive UC 7 years earlier. She had received multiple prior therapies for disease flares, including corticosteroids, IFX, adalimumab (ADA), golimumab, ustekinumab, and granulocyte and monocyte adsorptive apheresis (GMA), while being maintained on 5-aminosalicylic acid (5-ASA). She presented with another relapse characterized by >10 bloody stools per day, fever, and joint pain involving the right wrist. On admission, she had a body temperature of 37.2°C, a pulse rate of 78 beats/min, a hemoglobin level of 9.0 g/dL, and a C-reactive protein (CRP) level of 13.05 mg/dL. Based on frequent bloody stools, anemia, and elevated inflammatory markers, she was diagnosed with ASUC. Colonoscopy revealed extensive ulcers predominantly in the transverse colon (Fig. 1a, b).

**Fig. 1.**
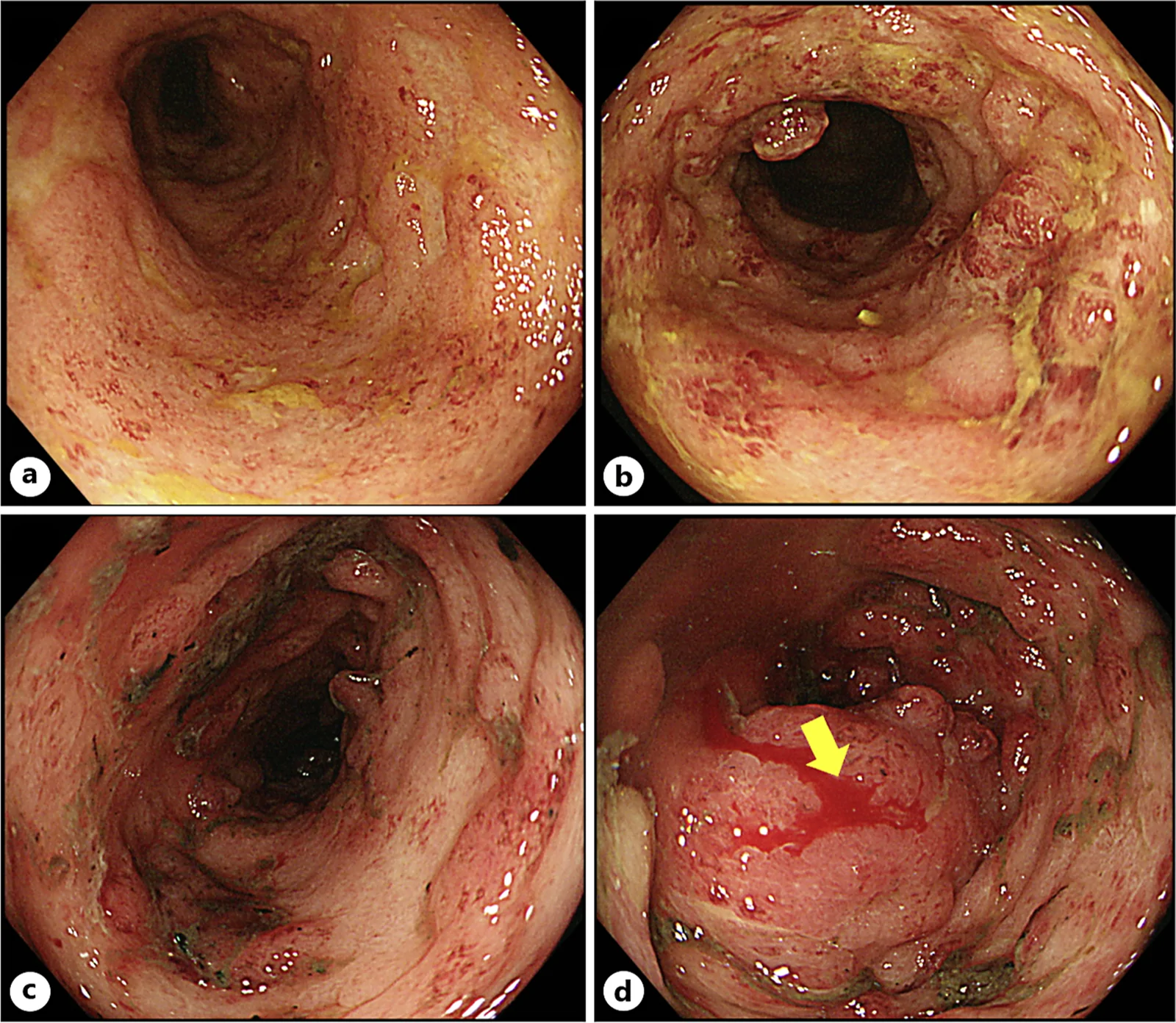
Colonoscopic findings in case 1. **a**, **b** At admission, colonoscopy showed extensive ulcers predominantly in the transverse colon, consistent with a Mayo endoscopic subscore of 3. **c**, **d** During a bleeding episode 6 days after UPA initiation, colonoscopy showed persistent oozing bleeding from the mucosa around epithelializing ulcers. Endoscopic hemostatic treatment with argon plasma coagulation was performed.

Given her previous steroid-refractory disease course, exposure to multiple biologic therapies, and the need for rapid disease control in ASUC, UPA was selected after surgical consultation. UPA 45 mg/day was initiated, while celecoxib was also started for joint pain. Stool frequency and hematochezia improved rapidly, and by 5 days after UPA initiation, the CRP level had decreased from 13.05 mg/dL at admission to 5.53 mg/dL. However, on the same day, she developed massive hematochezia, hypotension, and transient loss of consciousness. Her hemoglobin level decreased from 8.5 g/dL to 6.4 g/dL, requiring transfusion of 4 units of red blood cells. Emergency colonoscopy performed 6 days after UPA initiation demonstrated improvement of the ulceration, whereas persistent oozing was observed from the mucosa around epithelializing ulcers; therefore, endoscopic hemostatic treatment with argon plasma coagulation was performed (Fig. 1c, d). The bleeding sites did not correspond to the biopsy sites at admission.

Hematochezia recurred 7 days after UPA initiation, while the CRP level had further decreased to 1.13 mg/dL and stool frequency continued to improve. Her hemoglobin level decreased from 8.4 g/dL to 6.6 g/dL, requiring transfusion of 4 units of red blood cells. Nevertheless, hematochezia recurred again 12 days after UPA initiation, when the CRP level had normalized to 0.12 mg/dL. Colonoscopy revealed scattered oozing bleeding from multiple sites around epithelializing ulcers, and additional argon plasma coagulation was performed. Although stool frequency and inflammatory biomarkers had markedly improved, hematochezia and hypotension recurred for the fourth time 19 days after UPA initiation. Because the frequency and severity of bleeding became increasingly disproportionate to the marked improvement in UC symptoms and inflammatory biomarkers, UPA-associated bleeding was suspected. After discontinuation of UPA, no further bleeding occurred. Treatment was subsequently continued with GMA, and she was discharged.

### Case 2

A 39-year-old woman had been diagnosed with extensive UC 17 years earlier and had undergone multiple courses of remission induction with corticosteroids. She presented to a referring hospital with a relapse characterized by >10 bloody stools per day and abdominal pain. Colonoscopy performed at the referring hospital revealed deep ulcers extending from the ascending colon to the transverse colon (Fig. 2a, b). Intravenous corticosteroids and IFX were initiated at the referring hospital; however, her response was insufficient, and she was transferred to our hospital for further treatment. On admission to our hospital, she had persistent abdominal pain and >10 bloody stools per day. Her body temperature was 38.1°C, pulse rate was 80 beats/min, hemoglobin level was 13.2 g/dL, and CRP level was 10.63 mg/dL. Based on frequent bloody stools and an elevated CRP level, she was diagnosed with ASUC.

**Fig. 2.**
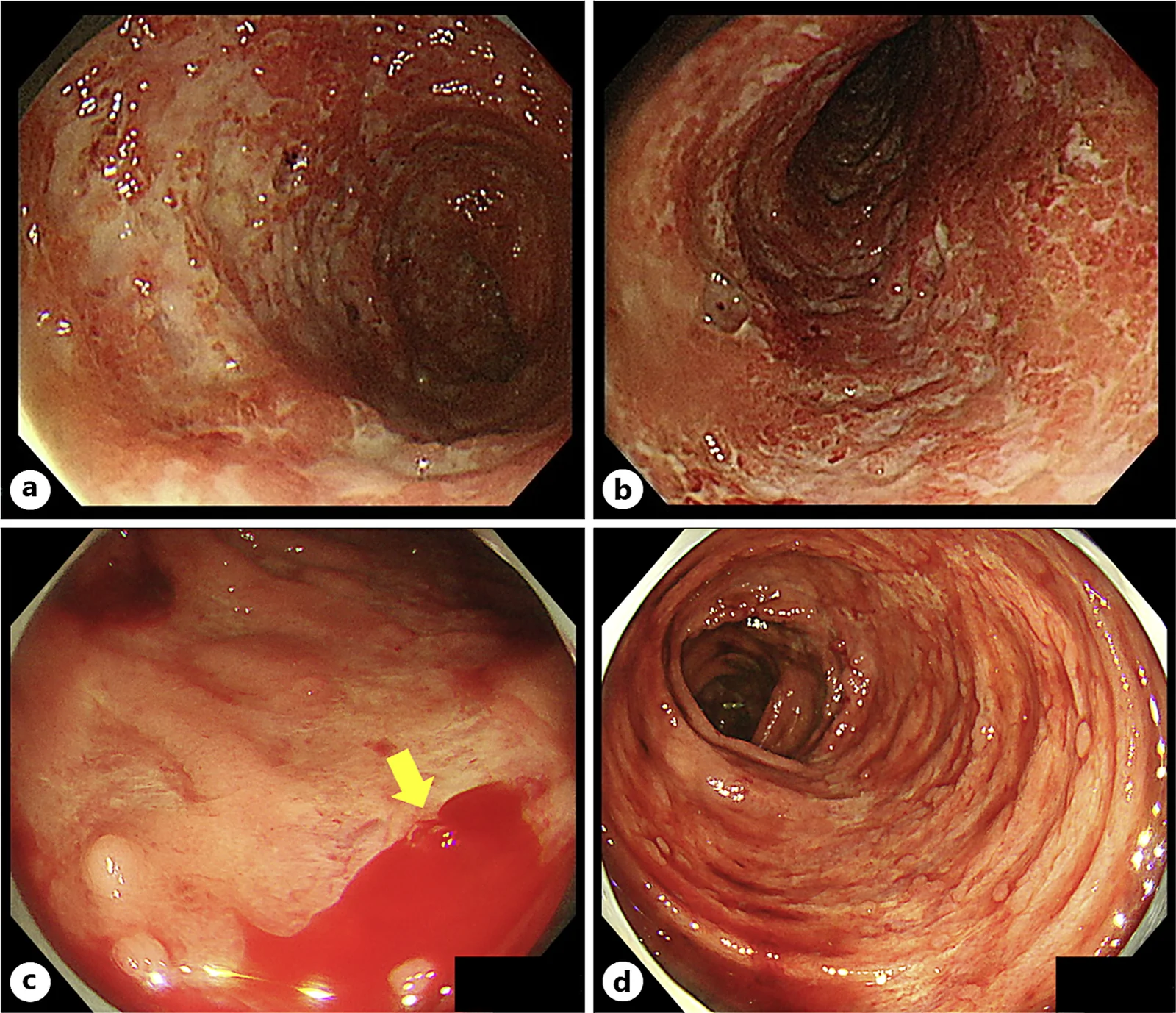
Colonoscopic findings in case 2. **a**, **b** Colonoscopy performed at the referring hospital showed extensive deep colonic ulcers, consistent with a Mayo endoscopic subscore of 3. **c**, **d** During a bleeding episode 10 days after UPA initiation, colonoscopy showed spurting bleeding from an exposed vessel at an epithelializing ulcer base in the transverse colon. Endoscopic hemostatic treatment with clipping was performed.

Because she had an insufficient response to intravenous corticosteroids and IFX at the referring hospital, UPA was selected as rescue therapy after surgical consultation. UPA 45 mg/day was initiated after transfer. After UPA initiation, stool frequency, hematochezia, and inflammatory markers rapidly improved. Hematochezia resolved 3 days after UPA initiation, and the CRP level decreased to 1.34 mg/dL on the same day. However, on the same day, she developed massive hematochezia, hypotension, and transient loss of consciousness. Her hemoglobin level decreased to 8.3 g/dL, and transfusion of 2 units of red blood cells was performed. Colonoscopy showed epithelialized ulcer bases, whereas spurting bleeding from an exposed vessel at an epithelializing ulcer base was observed in the ascending colon. Endoscopic hemostatic treatment with clipping was performed.

Seven days after UPA initiation, despite a decrease in the CRP level to 0.24 mg/dL, she again developed massive hematochezia, hypotension, and transient loss of consciousness. Colonoscopy demonstrated spurting bleeding from an exposed vessel at an epithelialized ulcer base in the cecum, despite further improvement in colonic inflammation, and additional endoscopic clipping was performed. Because recurrent bleeding was disproportionate to the improvement in UC activity, UPA-associated bleeding was suspected, and UPA was discontinued 9 days after UPA initiation. Ten days after UPA initiation, despite normalization of the CRP level to 0.06 mg/dL, hematochezia recurred. Colonoscopy showed further epithelialization of UC-related ulcers, whereas bleeding from an exposed vessel at an epithelialized ulcer base in the transverse colon was observed, and endoscopic clipping was performed (Fig. 2c, d). The bleeding sites corresponded to previously ulcerated areas in the ascending and transverse colon, but none corresponded to recent biopsy sites. Treatment was subsequently switched to mirikizumab, and no further bleeding occurred thereafter.

### Case 3

A 51-year-old man had been diagnosed with extensive UC 17 years earlier. He had previously been treated with prednisolone, IFX, ADA, and GMA. He had been maintained in remission with 5-ASA and 6-mercaptopurine (6-MP) but had been lost to follow-up for 2 years. He presented with abdominal pain and >10 bloody stools per day. On admission, he had a body temperature of 37.5°C, a pulse rate of 93 beats/min, a hemoglobin level of 15.5 g/dL, and a CRP level of 13.07 mg/dL. He was diagnosed with ASUC and admitted to our hospital. Colonoscopy revealed extensive ulcers predominantly in the ascending colon (Fig. 3a, b).

**Fig. 3.**
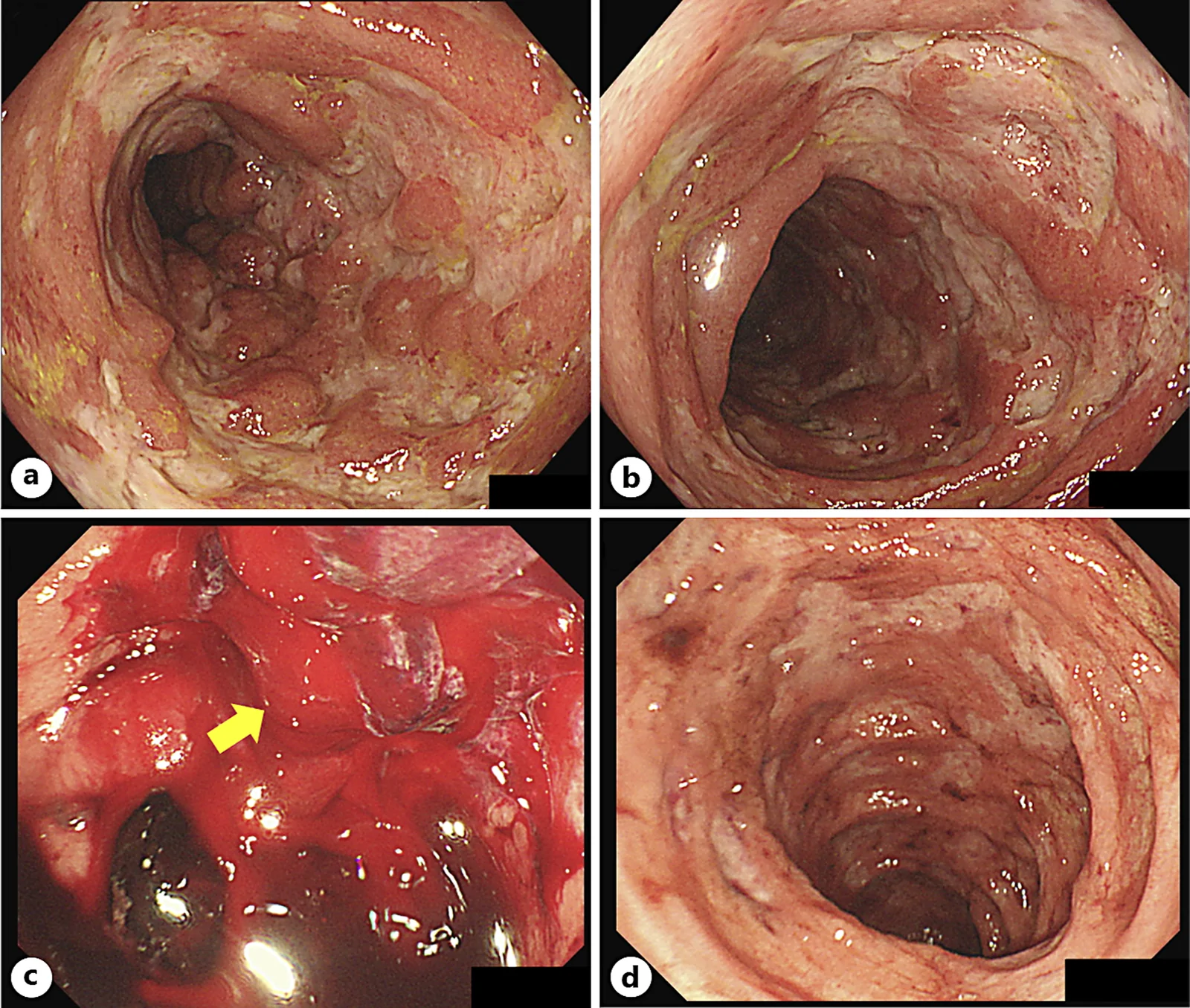
Colonoscopic findings in case 3. **a**, **b** At admission, colonoscopy showed extensive ulcers predominantly in the ascending colon, consistent with a Mayo endoscopic subscore of 3. **c**, **d** During a bleeding episode 7 days after UPA initiation, colonoscopy showed spurting bleeding from an exposed vessel at an epithelializing ulcer base in the ascending colon. Endoscopic hemostatic treatment with clipping was performed.

Given his prior exposure to IFX and the need for rapid disease control, UPA was selected after surgical consultation. UPA 45 mg/day was initiated. Nonsteroidal anti-inflammatory drugs (NSAIDs), flurbiprofen axetil, was also started for abdominal pain. Three days after UPA initiation, hematochezia had resolved and abdominal pain had improved; however, because the CRP level remained elevated at 10.31 mg/dL, prednisolone 40 mg/day was added. Thereafter, inflammatory markers improved, and 7 days after UPA initiation, the CRP level had decreased to 1.97 mg/dL. However, on the same day, he developed massive hematochezia, hypotension, and transient loss of consciousness. His hemoglobin level decreased from 10.3 g/dL to 7.2 g/dL, requiring transfusion of 4 units of red blood cells. Colonoscopy identified spurting bleeding from an exposed vessel at an epithelialized ulcer base in the ascending colon. Endoscopic hemostatic treatment with clipping was performed (Fig. 3c, d). The bleeding site corresponded to a previously ulcerated area in the ascending colon but did not correspond to a recent biopsy site. Considering the possibility of UPA-related bleeding, UPA was discontinued. Treatment was subsequently resumed with 5-ASA and 6-mercaptopurine. No further bleeding occurred thereafter, and he was discharged.

The clinical characteristics, treatment responses, and bleeding events in the 3 patients are summarized in Table 1. None of the patients were receiving antiplatelet or anticoagulant therapy at the time of bleeding. Platelet counts and coagulation parameters at the time of the bleeding episodes are summarized in Table 1. In all 3 patients, cytomegalovirus (CMV) colitis was excluded based on negative CMV antigenemia and the absence of CMV inclusion bodies on histopathological examination. *Clostridioides difficile* infection and other bacterial enterocolitis were also excluded in all 3 patients by negative stool testing and stool cultures, respectively.

**Table 1.** Clinical characteristics, treatment response, and bleeding events of the 3 cases

​	Case 1	Case 2	Case 3
Baseline characteristics			
Age, years	50	39	51
Sex	Female	Female	Male
Disease extent	Extensive colitis	Extensive colitis	Extensive colitis
Duration of disease, years	7	17	17
Prior biologics	IFX, ADA, GLM, UST	IFX	IFX, ADA
CRP at admission, mg/dL	13.05	10.63	13.07
PRO2 at admission	6	5	5
UPA response and bleeding timeline			
UPA dose at bleeding onset, mg/day	45	45	45
Timing of bleeding after UPA initiation	Days 5, 7, 12, 19	Days 3, 7, 10	Days 7
CRP at bleeding episodes, mg/dL	5.53 (Days 5)	1.34 (Days 3)	1.97 (Days 7)
	1.13 (Days 7)	0.24 (Days 7)	​
	0.12 (Days 12)	0.06 (Days 10)	​
	0.05 (Days 19)	​	​
PRO2 before first bleeding	3 (Days 4)	3 (Days 2)	3 (Days 6)
Bleeding characteristics and management			
Bleeding pattern	Oozing	Spurting	Spurting
Hemostatic treatment	APC	Clipping	Clipping
Timing of UPA discontinuation	Days 19	Days 9	Days 7
Bleeding after UPA discontinuation	No	Yes (Days 10)	No
Potential contributing factors			
Concomitant corticosteroid use at bleeding	No	Yes	Yes
Concomitant NSAID use at bleeding	Yes	No	Yes
Antiplatelet/anticoagulant use at bleeding	No	No	No
Platelet count at first bleeding, ×10^3^/μL	494	623	550
PT-INR at first bleeding	1.01	0.99	1.06
APTT at first bleeding, s	28.1	26.3	33.2

All time points after UPA initiation are expressed as days after the first UPA dose.

ADA, adalimumab; APC, argon plasma coagulation; APTT, activated partial thromboplastin time; CRP, C-reactive protein; GLM, golimumab; IFX, infliximab; NSAID, nonsteroidal anti-inflammatory drug; PRO2, 2-item patient-reported outcome; PT-INR, prothrombin time-international normalized ratio; UPA, upadacitinib; UST, ustekinumab.

## Discussion

In our patients, massive colonic bleeding occurred after UPA initiation despite clinical, biochemical, and endoscopic improvement in UC activity. These clinical courses suggest that worsening UC activity alone may not fully explain the bleeding events. To the best of our knowledge, this is the first case series describing massive colonic bleeding during early response to UPA in ASUC.

Rectal bleeding is one of the major symptoms of UC and generally reflects active mucosal inflammation and correlates with disease activity [12]. It has been reported that rectal bleeding could be improved as early as day 1 of induction treatment with UPA [13]. Given this rapid onset of action, UPA is increasingly used as rescue therapy for ASUC, as shown by recent multicenter studies and systematic reviews [8–10]. Massive bleeding may occur as a rare but recognized complication of ASUC with deep ulceration. However, to the best of our knowledge, massive bleeding requiring endoscopic hemostasis despite apparent improvement has not been reported in ASUC treated with conventional rescue therapies or other JAK inhibitors. Therefore, the bleeding observed in our cases may reflect a specific clinical context involving deep ulceration and early mucosal repair in ASUC.

Several adverse events associated with UPA, including infections, herpes zoster, acne, and laboratory abnormalities, have been reported with increased incidence in both clinical trials and real-world studies [7, 14]. Although gastrointestinal adverse events have not been commonly reported, worsening of the underlying disease has been described in clinical trials, and some of these cases were presumed to be accompanied by worsening gastrointestinal bleeding [7]. In addition, reports of gastrointestinal perforation during UPA treatment in patients with UC and other immune-mediated inflammatory diseases should not be overlooked [15, 16].

The mechanisms underlying these gastrointestinal adverse events associated with UPA have not been fully elucidated. However, changes in cytokine networks involved in intestinal barrier immunity may play a role. In rheumatoid arthritis, treatment with tocilizumab, an interleukin-6 (IL-6) inhibitor, has been associated with an increased risk of gastrointestinal perforation [17, 18]. UPA is a selective JAK1 inhibitor and suppresses JAK1-mediated signaling, including IL-6 signal transduction via the JAK-STAT pathway [19]. Therefore, modulation of IL-6-related signaling may contribute to gastrointestinal mucosal vulnerability during UPA treatment. In addition, bleeding in the present cases arose from regenerating mucosa or exposed vessels at epithelializing ulcer bases, suggesting that local vascular fragility during the early repair phase of deep ulceration may also have been involved. Angiogenesis and VEGF-related signaling are important for vascular repair, and a biomarker analysis of filgotinib, another JAK1-preferential inhibitor, showed reductions in VEGFA and other inflammatory biomarkers in patients with Crohn’s disease [20]. Thus, cytokine-mediated mucosal vulnerability and altered angiogenic or vascular repair pathways may both be involved in these bleeding events.

In the present case series, all patients developed bleeding during induction therapy with 45 mg/day of UPA while receiving corticosteroids, NSAIDs, or both. These agents have been identified as risk factors for lower gastrointestinal complications associated with IL-6 inhibitors in rheumatoid arthritis [17, 18]. These concomitant medications may have contributed to mucosal fragility in our cases, resulting in colonic bleeding. Another important point is that this type of bleeding may be difficult to distinguish from UC-related hematochezia. In our cases, the bleeding was initially considered to be related to residual UC activity in some patients, resulting in delayed discontinuation of UPA. Consequently, similar events in clinical trials may have been overlooked as worsening UC activity rather than recognized as potential treatment-related complications, especially in the blinded clinical trial settings during the induction phase.

When massive colonic bleeding occurs after UPA initiation in patients with ASUC despite clinical, biochemical, and endoscopic improvement, clinicians should consider that the bleeding may not necessarily reflect worsening of UC activity. Prompt endoscopic reassessment and reconsideration of treatment strategy, including whether UPA should be continued, may therefore be warranted. However, because all patients underwent endoscopic hemostatic treatment and case 2 experienced another bleeding episode after UPA discontinuation, the absence of subsequent recurrent bleeding may not be solely attributable to UPA withdrawal. These cases raise the possibility that massive colonic bleeding may occur in a specific clinical context, namely ASUC with vulnerable colonic mucosa, high-dose UPA induction therapy, and concomitant corticosteroid or NSAID use. Careful monitoring is warranted during the early response to UPA in patients with ASUC. However, the true incidence, risk factors, and mechanisms of this event remain unclear, and further accumulation of cases and pharmacovigilance data is warranted.

## Statement of Ethics

Ethical approval was not required for this case series in accordance with national and institutional guidelines. Written informed consent was obtained from all patients for publication of the details of their medical cases and any accompanying images.

## Conflict of Interest Statement

Kunio Asonuma has served as a speaker for EA Pharma Co., Ltd., Takeda Pharmaceutical Company Limited, Mitsubishi Tanabe Pharma Corporation, AbbVie Inc., Mochida Pharmaceutical Co., Ltd., Janssen and Pfizer. Akira Nogami has served as a speaker for AbbVie GK, Mochida Pharmaceutical Co. Taku Kobayashi reports serving as an advisory board member, consultant, or speaker for AbbVie GK, Alfresa Pharma Corporation, Alimentiv, Bristol Myers Squibb, Celltrion, Covidien, EA Pharma, Eli Lilly, Ferring Pharmaceuticals, Gilead Sciences, Janssen Pharmaceutical, JIMRO, Kissei Pharmaceutical, Kowa, Kyorin Pharmaceutical, Mitsubishi Tanabe Pharma Corporation, Mochida Pharmaceutical, MSD, Nippon Kayaku, Pfizer, Sanofi, Sekisui Medical, Spyre, Takeda Pharmaceutical, TOPPAN Holdings, and Zeria Pharmaceutical and receiving research funding from AbbVie GK, Alfresa Pharma Corporation, Bristol Myers Squibb, EA Pharma, Gilead Sciences, Helmsley Charitable Trust, JIMRO, Kyorin Pharmaceutical, Miyarisan Pharmaceutical, Mochida Pharmaceutical, Nippon Kayaku, Pfizer, Sekisui Medical, Samsung Medison, Takeda Pharmaceutical, and Zeria Pharmaceutical. Taku Kobayashi was a member of the journal’s Editorial Board at the time of submission. The other authors have no conflicts of interest to declare.

## Funding Sources

This study was not supported by any sponsor or funder.

## Author Contributions

Keita Suzuki collected the clinical data and drafted the manuscript. Kunio Asonuma, Akira Nogami, Kanade Serizawa, and Kenta Nakamura contributed to patient management and interpretation of clinical data. Taku Kobayashi supervised the study and critically revised the manuscript. All authors read and approved the final version of the manuscript.

## Data Availability

The data supporting the findings of this study are not publicly available because they contain information that could compromise the privacy of the patients but are available from the corresponding author upon reasonable request.
